# 2953. PK/PD Comparison of a New Dosing Scheme for Meropenem Adapted to Outpatient Parenteral Antimicrobial Therapy (OPAT) in Non-Critically-Ill Patients

**DOI:** 10.1093/ofid/ofad500.192

**Published:** 2023-11-27

**Authors:** Caroline Briquet, Hospital Pharmacist, Perrin Ngougni Pokem, Gert-Jan Wijnant, Olivier Cornu, Halil Yildiz, Françoise Van Bambeke, Jean Cyr Yombi

**Affiliations:** Cliniques Universitaires Saint-Luc - UCLouvain - Brussels - Belgium, Brussels, Brussels Hoofdstedelijk Gewest, Belgium; UCLouvain - Brussels - Belgium, Brussels, Brussels Hoofdstedelijk Gewest, Belgium; UCLouvain - Brussels - Belgium, Brussels, Brussels Hoofdstedelijk Gewest, Belgium; Cliniques Universitaires Saint-Luc - UCLouvain - Brussels - Belgium, Brussels, Brussels Hoofdstedelijk Gewest, Belgium; Cliniques Universitaires Saint-Luc - UCLouvain - Brussels - Belgium, Brussels, Brussels Hoofdstedelijk Gewest, Belgium; UCLouvain - Brussels - Belgium, Brussels, Brussels Hoofdstedelijk Gewest, Belgium; Cliniques Universitaires Saint-Luc - UCLouvain - Brussels - Belgium, Brussels, Brussels Hoofdstedelijk Gewest, Belgium

## Abstract

**Background:**

A pilot OPAT project has been started in Belgium for the continuation at home of the treatment (initiated in hospital) of patients infected by multi-resistant Gram-negative bacteria when no oral options are available. In Belgium, meropenem is the only available carbapenem, but its limited stability at room temperature does not allow a sustained administration over 12 or 24 h via IV pumps. As the replacement of the perfusion is not feasible after working hours for home care nurses, the aim of this prospective study was to compare the pharmacokinetic profile and time above MIC for 2g meropenem administered TID (20 minutes infusion) over 12h (typically at 8h,14h, 20h) in comparison with the traditional 24h regimen (typically at 8h,16h, 24h).

**Figure:**
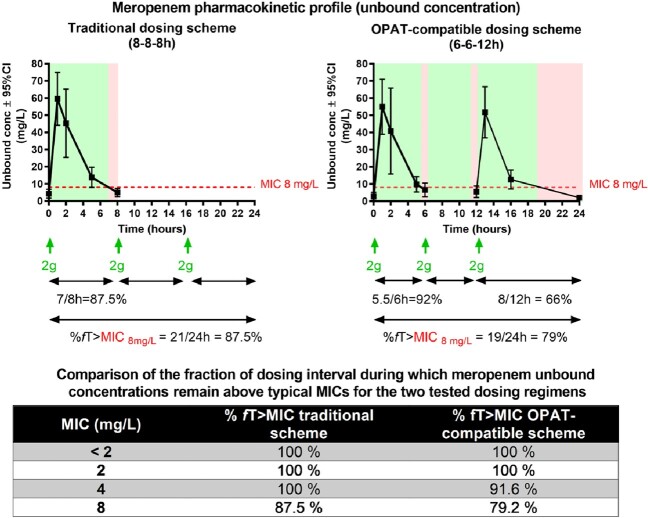

**Methods:**

Patients received first meropenem 2g q8h. At steady state ( > 3 doses), 4 blood samples were collected over 8h. The penultimate day of the treatment, patients were switched to the OPAT-compatible dosage regimen (2g q6h over 12h) and, at steady state, a total of 8 blood samples were collected during the first and third dosing intervals. Unbound meropenem concentrations were determined in ultrafiltrated plasma by a validated LC-MS/MS procedure. Time above MICs ≤ 8 mg/L (EUCAST susceptibility breakpoint) were estimated for both schemes based on PK profiles.

**Results:**

Twenty-two patients were included. The mean pharmacokinetic profile for each scheme is shown in the Figure 1. The Table 1 shows that plasma concentrations remained > 4 mg/L during 100% time with the conventional scheme and > 2 mg/L with the OPAT-compatible scheme but still at least ∼80% time > 8 mg/L with both therapeutic schemes. Periods of inadequate coverage are spread at the end of each dose with the conventional dosing regimen but essentially concentrated overnight with the new scheme.

**Conclusion:**

PK/PD models recommend for meropenem therapeutic schemes with plasma concentrations > MIC during 30-40% of the time in non-critically ill patients. This target is easily achieved with the proposed OPAT-compatible dosing regimen, including during night, and could therefore be safely used in a context of limited nurse resources.

**Disclosures:**

**All Authors**: No reported disclosures

